# Microgravity and Cellular Biology: Insights into Cellular Responses and Implications for Human Health

**DOI:** 10.3390/ijms26073058

**Published:** 2025-03-27

**Authors:** Nelson Adolfo López Garzón, María Virginia Pinzón-Fernández, Jhan S. Saavedra T., Humberto A. Nati-Castillo, Marlon Arias-Intriago, Camila Salazar-Santoliva, Juan S. Izquierdo-Condoy

**Affiliations:** 1 Grupo de Investigación en Salud (GIS), Departamento de Medicina Interna, Universidad del Cauca, Popayan 190003, Colombia; 2 Interinstitutional Internal Medicine Group (GIMI 1), Department of Internal Medicine, Universidad Libre, Cali 760031, Colombia; 3 One Health Research Group, Universidad de las Américas, Quito 170124, Ecuador

**Keywords:** microgravity, tissue effects, immune system, cardiomyocytes, cancer biology, human health

## Abstract

Microgravity, defined by minimal gravitational forces, represents a unique environment that profoundly influences biological systems, including human cells. This review examines the effects of microgravity on biological processes and their implications for human health. Microgravity significantly impacts the immune system by disrupting key mechanisms, such as T cell activation, cytokine production, and macrophage differentiation, leading to increased susceptibility to infections. In cancer biology, it promotes the formation of spheroids in cancer stem cells and thyroid cancer cells, which closely mimic in vivo tumor dynamics, providing novel insights for oncology research. Additionally, microgravity enhances tissue regeneration by modulating critical pathways, including Hippo and PI3K-Akt, thereby improving stem cell differentiation into hematopoietic and cardiomyocyte lineages. At the organ level, microgravity induces notable changes in hepatic metabolism, endothelial function, and bone mechanotransduction, contributing to lipid dysregulation, vascular remodeling, and accelerated bone loss. Notably, cardiomyocytes derived from human pluripotent stem cells and cultured under microgravity exhibit enhanced mitochondrial biogenesis, improved calcium handling, and advanced structural maturation, including increased sarcomere length and nuclear eccentricity. These advancements enable the development of functional cardiomyocytes, presenting promising therapeutic opportunities for treating cardiac diseases, such as myocardial infarctions. These findings underscore the dual implications of microgravity for space medicine and terrestrial health. They highlight its potential to drive advances in regenerative therapies, oncology, and immunological interventions. Continued research into the biological effects of microgravity is essential for protecting astronaut health during prolonged space missions and fostering biomedical innovations with transformative applications on Earth.

## 1. Introduction

Microgravity, a condition characterized by minimal and almost imperceptible gravitational forces, occurs primarily in space or in specialized environments designed to simulate this state [1,2]. In cellular biology, microgravity presents a unique opportunity to study how cells respond to the drastic reduction in gravity—a fundamental factor that regulates numerous cellular processes on Earth [3,4,5]. Under microgravity conditions, mechanical forces that influence cell structure and function undergo significant alterations, impacting cytoskeletal organization, cell adhesion, and intracellular signaling. These changes in turn affect critical cellular processes, including proliferation, differentiation, communication, and survival [4,5,6].

Historically, research on microgravity has been conducted aboard space stations, such as the International Space Station, and on suborbital platforms. More recently, terrestrial laboratories have adopted advanced technologies, such as rotating bioreactors, to simulate microgravity conditions [1,7,8]. These investigations, conducted both in space and on Earth, have gained importance due to their dual applications: safeguarding astronaut health and addressing terrestrial diseases [1,9,10].

This review aims to explore recent advances in understanding the effects of microgravity on biological processes, emphasizing its implications and applications for human health.

## 2. Materials and Methods

A comprehensive literature review was conducted using PubMed, Scopus, and Embase databases. Search terms were structured following the PICOT framework, with the components defined as follows:

- Population: Stem cells, T cells, endothelial cells, cancer cells, kidney cells, and bone cells.
- Intervention: Microgravity, simulated microgravity, or microgravity conditions.
- Comparison: Cells under normal or standard gravity conditions.
- Outcomes: Effects on cell proliferation, tissue regeneration, cellular metabolism, organ homeostasis, and immune health.
- Timing: Publications with no start date restriction, available up to November 2024.

The research question, based on the PICO framework, was as follows:

*“How does microgravity (including simulated conditions) affect stem cells, T cells, endothelial cells, kidney cells, and other biological cells compared to cells under normal gravity conditions, specifically in terms of cell proliferation, tissue regeneration, cellular metabolism, organ homeostasis, and immune health?”*

To ensure a comprehensive search, relevant Medical Subject Headings (MeSH) terms were included. Key terms covered the study populations (e.g., stem cells, T cells, endothelial cells, cancer cells, kidney cells, bone cells), conditions (e.g., microgravity, simulated microgravity, standard gravity), and outcomes of interest (e.g., cell proliferation, tissue regeneration, cellular metabolism, organ homeostasis, immune health).

The literature search was performed in December 2024 and included publications in English and Spanish. Eligible studies encompassed observational studies, in vitro research, and experimental investigations that addressed the effects of microgravity on critical biological processes. The retrieved results were systematically categorized based on their focus, enabling a detailed analysis of microgravity’s impact on different biological functions. The findings were then contrasted with observations from cells maintained under standard gravity conditions, providing a comprehensive comparison of cellular adaptations in these environments.

## 3. Effects of Microgravity on the Immune System

### 3.1. Alterations in Immune Cells

Microgravity significantly impacts the immune system, compromising the development, function, and response of immune cells, which weakens the body’s defenses in space environments. Hematopoietic stem cells, essential for generating immune cells, show impaired differentiation into macrophages, critical for infection control. Furthermore, an imbalance between M1 (pro-inflammatory) and M2 (anti-inflammatory and repair) macrophages disrupts the immune system’s ability to handle infections and repair tissues [1,2].

Dendritic cells, pivotal in T lymphocyte activation via antigen presentation, are also adversely affected. Key molecules such as the major histocompatibility complex (MHC) and costimulatory proteins CD80 and CD86 show reduced expression, diminishing dendritic cell functionality. Notably, brief exposures to microgravity transiently enhance dendritic cell activity; however, this effect is not sustained over the long term [2].

**Figure 1:**
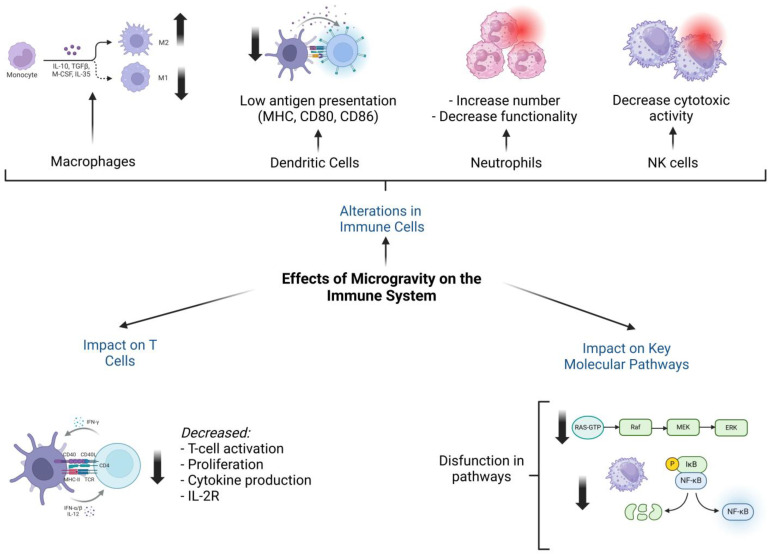
Effects of microgravity on the human immune system. This figure illustrates the complex impact of microgravity on various immune cell populations and key molecular signaling pathways. Exposure to microgravity leads to significant alterations in immune cell function, including a shift in macrophage polarization from the pro-inflammatory M1 phenotype to the anti-inflammatory M2 phenotype. Dendritic cells exhibit a reduced capacity for antigen presentation, while neutrophil numbers increase, but their functionality declines. Additionally, the cytotoxic activity of natural killer (NK) cells is diminished, potentially impairing immune surveillance. T cell activation, proliferation, and cytokine production are also suppressed, accompanied by decreased expression of IL-2R. At the molecular level, microgravity disrupts critical signaling pathways, including the RAS-RAF-MEK-ERK cascade and the NF-κB pathway, further contributing to immune dysregulation. These findings highlight the widespread and multifaceted effects of microgravity on immune system function, which may have implications for long-term space travel and astronaut health.

Neutrophils exhibit increased numbers in microgravity but with reduced functionality, impairing their ability to destroy microorganisms effectively. B lymphocytes, responsible for humoral immunity, demonstrate decreased proliferation and increased apoptosis. Similarly, natural killer (NK) cells, critical for innate immunity, experience reduced cytotoxic activity, limiting their ability to eliminate tumors or infected cells [2,3] (Figure 1).

### 3.2. Impact on T Cells

Microgravity profoundly affects T cell-mediated immunity, disrupting activation, proliferation, and apoptosis. Studies using concanavalin A—a standard activator of resting T cells—revealed significant inhibition of activation and proliferation in CD4+ and CD8+ T cells, with the effects intensifying over longer exposure durations. Reduced expression of activation markers such as CD25 (IL-2 receptor), CD69 (early activation marker), and CD71 (iron transport marker) underscores this dysfunction [11,12].

CD4+ T cells, critical for regulating immune responses through cytokines like IL-2 and IFN-γ, show a greater reduction in proliferation and cytokine production compared to CD8+ T cells. Even brief exposure (8 h) to microgravity notably suppresses CD4+ cell functions, while CD8+ cells require longer exposure (16 h) to exhibit similar impairments [11,12].

Cytokine production is notably compromised under microgravity. CD4+ cells exhibit decreased secretion of IL-2, essential for T cell expansion, and IFN-γ, crucial for macrophage activation and antiviral defense. These deficiencies indicate a reduced capacity of T cells to coordinate and amplify immune responses [11].

Apoptosis of T cells is accelerated under microgravity, with an earlier onset of late apoptotic processes. Pre-exposed cells show an increase in late-stage apoptosis and a reduction in early-stage apoptosis, indicating a faster progression through apoptotic pathways compared to controls under normal gravity. This effect is linked to increased expression of the Fas receptor in CD8+ cells, a critical component of the Fas/FasL pathway, which regulates apoptosis through external signals [12,13,14] (Figure 1).

### 3.3. Impact on Key Molecular Pathways

At the molecular level, microgravity disrupts critical signaling pathways, including RAS, ERK, and NF-κB. The RAS pathway, which governs cell proliferation and survival, shows reduced activity under microgravity, leading to impaired cell growth. Similarly, ERK, which is essential for cell differentiation and responses to external stimuli, undergoes alterations that hinder the activation and specialization of macrophages and lymphocytes. NF-κB, a key regulator of inflammatory responses, exhibits inconsistent activation in microgravity, resulting in inadequate immune responses [1,4].

Research on microgravity has also revealed significant effects on cancer biology, including the formation of multicellular spheroids (MCS), the reorganization of the cytoskeleton, and the regulation of key proteins such as p53 (a tumor suppressor), NF-κB (a regulator of immune responses and inflammation), and BAX (a pro-apoptotic protein involved in programmed cell death). These processes promote apoptosis in various cancer cell types. In cell lines from breast (MCF-7), thyroid (FTC-133), and lung (A549) cancers, microgravity-induced changes include decreased cell adhesion and altered gene expression, particularly in molecules like E-cadherin (important for cell adhesion and tumor invasion suppression), vascular endothelial growth factor (VEGF, which promotes angiogenesis), and vimentin (a protein associated with epithelial–mesenchymal transition and cell migration), thereby affecting tumor proliferation and dissemination [15,16].

## 4. Impact on Cancer Biology

### 4.1. Cancer Stem Cells

The study of cancer stem cells (CSCs) under simulated microgravity conditions provides critical insights into their behavior in extreme environments, with profound implications for oncology and regenerative medicine. CSCs, characterized by their self-renewal and differentiation capabilities, contribute to tumor heterogeneity, treatment resistance, and metastasis [5,6].

In cancers such as breast, lung, and gastrointestinal cancer, CSCs are identified using specific markers, enabling their isolation and detailed analysis. Under simulated microgravity, these cells exhibit significant changes in morphology and growth patterns, including the formation of multicellular spheroids. These three-dimensional structures closely mimic in vivo tumor organization, offering a robust model for studying the effects of microgravity on tumor biology [5,7,8].

**Figure 2:**
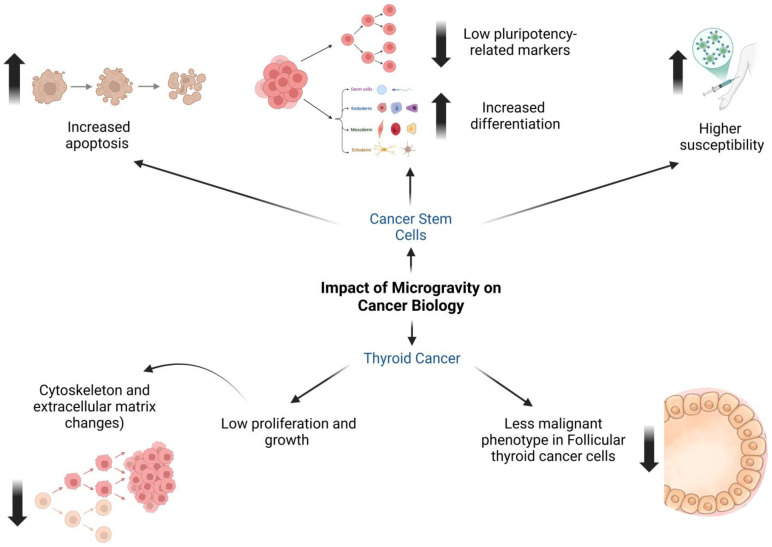
Microgravity-induced changes in cancer. This figure illustrates the diverse effects of microgravity on cancer cells, with a particular focus on cancer stem cells (CSCs) and thyroid cancer. Exposure to microgravity has been shown to increase apoptosis, or programmed cell death, in cancer cells. It also alters CSC properties, leading to a decrease in pluripotency markers and an increase in differentiation. Additionally, microgravity induces structural changes in the cytoskeleton and extracellular matrix, which may influence tumor progression. In thyroid cancer, microgravity reduces proliferation and overall growth, while also diminishing the malignant phenotype in follicular thyroid cancer cells. Interestingly, despite these inhibitory effects, microgravity appears to increase the susceptibility of cancer cells, potentially enhancing their response to treatments, as suggested by the syringe graphic. These findings highlight the complex and sometimes counterintuitive influence of microgravity on cancer biology.

Exposure to microgravity induces notable cellular changes, including increased apoptosis and decreased expression of pluripotency-related markers, suggesting a shift toward differentiation. This differentiation reduces CSC resistance, potentially enhancing their susceptibility to treatments. In gastrointestinal cancers like colon and pancreatic cancer, simulated microgravity has been linked to increased expression of specific markers, reflecting alterations in the regulatory mechanisms of cell growth and differentiation. These changes can significantly influence tumor progression and the efficacy of oncological treatments [6,7,8] (Figure 2).

Microgravity modulates apoptosis, a key defense against abnormal cell proliferation. Many cancer cells under microgravity show increased apoptosis due to altered mechanical forces, while some acquire stem-like properties, enhancing resistance, plasticity, and therapy resistance [17,18]. Microgravity also induces the formation of multicellular spheroids (MCS), which mimic solid tumors and exhibit increased resistance to chemotherapy and radiation, suggesting more aggressive cancer states. Additionally, reduced gravity downregulates adhesion proteins (e.g., E-cadherin, vinculin, paxillin), weakening cell–cell interactions and potentially enhancing metastatic potential. The combination of microgravity and ionizing radiation exacerbates DNA damage, genomic instability, and mutations in tumor cells, highlighting the need for protective measures during space missions and offering insights into cancer treatment [19,20,21,22].

Simulated microgravity, using devices like the rotating wall bioreactor (RWV) and random positioning machine (RPM), replicates certain effects of real microgravity (r-µg) but with some differences. High-energy radiation with high linear energy transfer (high LET) increases genomic instability and mutation risks, promoting apoptosis and chromosomal aberrations [23,24].

In gliomas (U251 model), microgravity reduces the activity of focal adhesion kinase (FAK), RhoA, and Nek2 kinase, inhibiting tumor cell migration. Immune cells, such as T lymphocytes and macrophages, show decreased functionality, compromising immunosurveillance and hindering cancer detection [25,26,27].

Recent findings suggest that microgravity could offer therapeutic benefits by inhibiting NF-κB, reducing cancer spread, and decreasing VEGF activity to limit tumor growth through reduced angiogenesis. Changes in the Wnt/β-catenin pathway may slow tumor growth and metastasis. CSCs, typically resistant to therapies, become more vulnerable under microgravity, which also alters extracellular vesicle (EV) protein and microRNA content, opening opportunities for new biomarkers and targeted therapies [28].

The mechanosensitive channel ELKIN1 plays a crucial role in cellular responses to microgravity. Microgravity reduces tumor cell adhesion and invasion, offering potential therapeutic benefits, but these effects are ELKIN1-dependent. Its absence preserves cell structure and adhesion stability in microgravity, protecting healthy cells during prolonged exposure, but may enhance the persistence of cancer cells, limiting therapy effectiveness [29].

At the molecular level, microgravity reduces the nuclear localization of the transcriptional regulator YAP1, limiting excessive cell proliferation and invasiveness. This effect depends on ELKIN1 expression; its absence could lead to resistance or diminished responsiveness to mechanical stimuli. ELKIN1-mediated reduction in focal adhesion proteins may limit tumor aggressiveness but could pose a structural disadvantage for healthy tissues during extended space missions [29].

### 4.2. Thyroid Cancer

Microgravity, whether experienced in space or simulated on Earth, provides a unique experimental environment that has greatly advanced our understanding of cancer biology, particularly thyroid cancer [30,31,32,33]. For nearly two decades, studies on thyroid cancer cells under microgravity have yielded valuable insights into tumor behavior that are difficult to achieve under conventional laboratory conditions.

In microgravity, adherent thyroid cancer cells lose their adhesion properties and organize into multicellular spheroids. These structures closely resemble the physiological characteristics of tumors in vivo, making them ideal models for studying metastasis and evaluating novel targeted therapies [30,31].

Microgravity also affects fundamental cellular processes, including apoptosis, cytoskeletal organization, and extracellular matrix dynamics, which are critical for cell growth and proliferation. Notably, follicular thyroid cancer cells exposed to microgravity have demonstrated a tendency to adopt a less malignant phenotype. This suggests that microgravity conditions may induce favorable changes, potentially aiding in the treatment of thyroid cancer [30,31].

The findings from these studies have the potential to revolutionize traditional oncology research approaches. They facilitate the identification of key cellular alterations in cancer pathogenesis and support the development of innovative therapies aimed at improving patient outcomes. Furthermore, these investigations pave the way for novel preventive strategies and more effective treatments based on the unique cellular properties observed under microgravity [30,32].

## 5. Impacts on Tissues and Organ Systems

### 5.1. Microvascular Endothelial Cells

Simulated microgravity profoundly affects microvascular endothelial cells, which play a pivotal role in vascular homeostasis by regulating inflammation, angiogenesis, and vascular permeability [9,10].

Under normal gravity, endothelial cells respond to mechanical and chemical stimuli to preserve vascular function. However, under simulated microgravity, significant alterations are observed. One prominent effect is the inhibition of cell proliferation, which delays the repair of damaged blood vessels. Reduced production of interleukin-6 (IL-6), a cytokine crucial for the inflammatory response, further compromises immune functionality and vascular repair capacity [9,23].

Interestingly, microgravity stimulates the synthesis of nitric oxide, a critical mediator for blood vessel dilation and angiogenesis. This effect is linked to the activation of endothelial nitric oxide synthase (eNOS). While nitric oxide promotes vascular adaptation, its dysregulation can lead to pathological states, depending on the physiological context [9,23].

Research on simulated microgravity has also revealed significant effects on endothelial cell integrity and function. In one study, human umbilical vein endothelial cells, essential regulators of cardiovascular homeostasis, were exposed to simulated microgravity for up to 72 h using an RPM, which continuously rotates to nullify Earth’s gravitational vector [24].

After 24 h, notable changes in cellular biomechanical properties were observed, including a marked reduction in cell stiffness (Young’s modulus) and viscosity. Specifically, cellular elasticity decreased by approximately 30%, reaching critical levels with a 75% reduction after 72 h. This loss of rigidity makes endothelial cells more vulnerable to external hemodynamic stress, potentially impairing vascular integrity and increasing the risk of endothelial dysfunction [23,24].

Additionally, endothelial cells exhibited morphological changes under simulated microgravity conditions. Normally elongated and spindle-shaped, cells adopted a distinctly rounded phenotype after 24 h. The increase in circularity—more than three times greater than that of control cells—indicates significant cytoskeletal reorganization, which could affect vascular permeability and the cells’ ability to respond to mechanical stress over time [23,34].

**Figure 3:**
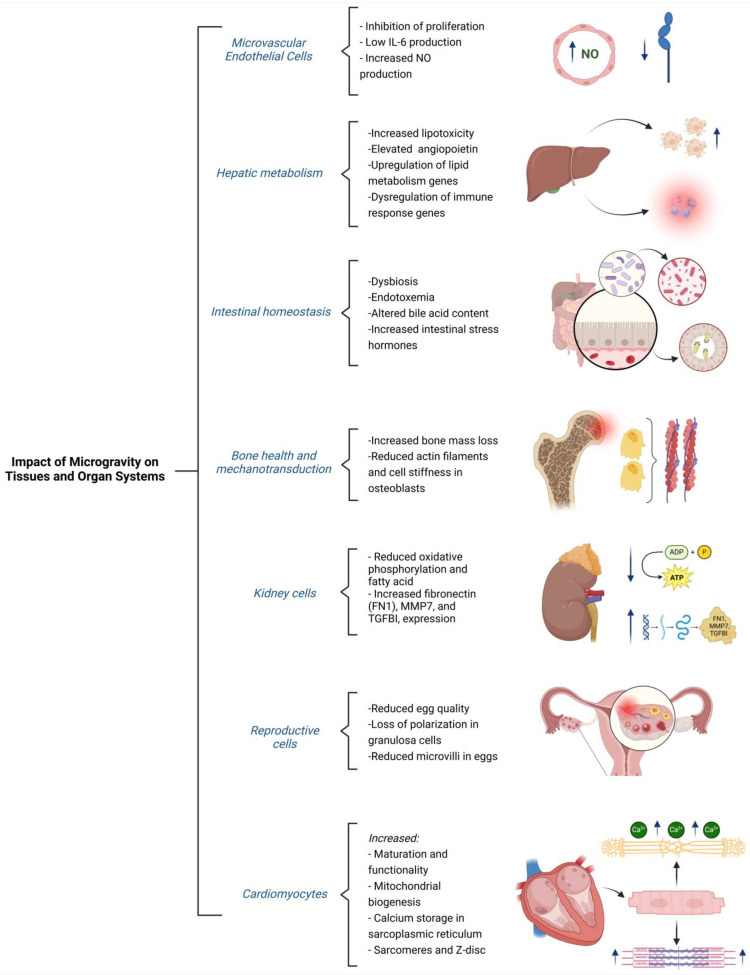
Structural and functional adaptations of tissues and organ systems in microgravity environments. This figure illustrates the broad impact of microgravity on various tissues and organ systems, highlighting key structural and functional adaptations. Microvascular endothelial cells exhibit inhibited proliferation, decreased IL-6 production, and increased nitric oxide (NO) production. In the liver, microgravity alters hepatic metabolism, leading to increased lipotoxicity, elevated angiopoietin levels, upregulation of lipid metabolism genes, and dysregulation of immune response genes. Intestinal homeostasis is also affected, with observed dysbiosis, endotoxemia, altered bile acid content, and increased levels of intestinal stress hormones. In terms of bone health and mechanotransduction, microgravity induces significant bone mass loss, accompanied by a reduction in actin filaments and decreased cell stiffness in osteoblasts. Kidney cells experience metabolic alterations, including reduced oxidative phosphorylation and fatty acid oxidation, alongside increased expression of fibronectin (FN1), MMP7, and TGFBI. Reproductive cells are also impacted, with evidence of reduced egg quality, the loss of polarization in granulosa cells, and a decrease in microvilli in eggs. Additionally, cardiomyocytes exhibit increased maturation and functionality, enhanced mitochondrial biogenesis, improved calcium storage in the sarcoplasmic reticulum, and the formation of sarcomeres and Z-disks. These findings collectively underscore the widespread and system-specific alterations induced by microgravity, significantly influencing cellular function and overall tissue homeostasis.

At the molecular level, these morphological alterations were associated with substantial reductions in key cytoskeletal components. Western blot analysis revealed a 65% reduction in actin filament levels and a 26% reduction in β-tubulin, a protein critical for microtubule integrity. Immunofluorescence confirmed these findings, showing discontinuities in cortical actin filaments and abnormal redistribution of microtubules, particularly around the nucleus. These results highlight impaired structural integrity and suggest that endothelial cells may have a compromised ability to respond to biomechanical stimuli [23,34] (Figure 3).

Endothelial cells exposed to microgravity also form three-dimensional spheroids, which closely mimic in vivo blood vessel structures. These 3D models are invaluable for studying microgravity-induced changes in endothelial function, enabling more physiologically relevant investigations compared to traditional 2D cultures [9,10,23].

Collectively, these findings reveal that microgravity disrupts endothelial physiology, influencing cell proliferation, inflammatory responses, and vascular regulation. These insights have implications for diseases such as cancer, atherosclerosis, and other circulatory disorders. They underscore the need for further research to mitigate vascular risks during space missions and explore potential therapeutic applications on Earth [9].

### 5.2. Hepatic Metabolism and Intestinal Homeostasis

Microgravity, a defining feature of spaceflight and terrestrial simulations, profoundly impacts the metabolism and functionality of various organs, particularly the liver. Experimental models using rhesus macaques have shown that prolonged exposure to microgravity induces significant metabolic alterations similar to those observed in rodents. These include lipid accumulation and infiltration of inflammatory cells in the liver, reflecting the disruption of hepatic metabolic homeostasis. Such findings underscore the importance of exploring the effects of microgravity on metabolic regulation in animal models and future human studies [34,35,36] (Figure 3).

In a 42-day head-down bed rest model simulating microgravity, rhesus macaques developed mild liver lesions, fat vacuole accumulation, and inflammatory cell infiltration. These hepatic changes were accompanied by a marked increase in plasma levels of angiopoietin-like 3 (ANGPTL3), a key biomarker associated with metabolic disorders and lipid dysregulation. These results suggest that microgravity may induce hepatic lipotoxicity, a phenomenon previously documented in rodents but requiring further investigation in primates and humans to better understand its clinical relevance [34,35,36].

At the molecular level, transcriptomic analyses of liver samples from macaques exposed to microgravity revealed overexpression of genes associated with lipid metabolism and downregulation of genes linked to the innate immune response. These molecular changes could impair the liver’s ability to manage metabolic stress, a critical function for maintaining long-term health. Additionally, disruptions in fatty acid metabolism were identified, potentially contributing to lipid dysregulation associated with metabolic diseases such as diabetes and obesity [34,35].

Another noteworthy finding was the impact of microgravity on the gut microbiota, a key regulator of hepatic metabolism through the gut–liver axis. Macaques subjected to the head-down bed rest model exhibited intestinal dysbiosis, endotoxemia, and altered bile acid composition. These gut alterations were accompanied by elevated levels of stress hormones, suggesting systemic metabolic imbalance triggered by microgravity exposure [34,36].

The effects of microgravity on liver function and metabolic homeostasis carry significant implications for space medicine. These findings highlight the need to closely monitor liver function and the gut–liver axis during prolonged space missions to mitigate potential health risks. Moreover, the metabolic changes observed in animal models provide valuable insights into terrestrial diseases such as diabetes and nonalcoholic fatty liver disease. This knowledge could contribute to the development of more effective treatments and innovative therapeutic strategies in regenerative medicine, ultimately benefiting both astronauts and the general population [34,35,36].

### 5.3. Bone Health and Mechanotransduction

Microgravity profoundly impacts the mechanotransduction of human osteoblasts. Astronauts who spend prolonged periods in space experience accelerated bone mass loss, making the International Space Station (ISS) an ideal platform for studying the mechanisms underlying osteoporosis in zero-gravity conditions [37,38,39].

In this context, innovative experimental approaches have been developed, including the use of microfluidic microchips and collagen-encapsulated cell spheroids. These advanced models enable researchers to investigate how the absence of mechanical loading affects bone cell maturation and mineralization. Studies have shown that microgravity decreases cell stiffness and reduces the levels of filamentous actin, a cytoskeletal component critical for maintaining cell tension and facilitating mechanotransduction. For example, experiments conducted on the ISS using automated microfluidic devices have provided valuable insights into the mechanical properties of human osteoblasts under microgravity conditions [37,38,39] (Figure 3).

Results from these experiments reveal that osteoblasts exposed to microgravity develop elongated protrusions compared to terrestrial samples, indicating a phenomenon referred to as “cellular softening”. This structural change correlates with a reduction in actin fiber density, consistent with findings from prior studies conducted under simulated microgravity [40,41,42].

These structural and cytoskeletal alterations highlight disruptions in mechanotransduction, the process through which cells sense and respond to mechanical stimuli. Without the constant gravitational forces present on Earth, osteoblasts lose the ability to maintain their tensional structure and perform optimally, contributing to the bone demineralization observed in astronauts [38,39].

Further research has utilized three-dimensional spheroid models, which better replicate physiological conditions than traditional two-dimensional cell cultures. Collagen-encapsulated osteoblast spheroids subjected to controlled pressures under microgravity exhibited a reduction in the expression of key mechanosensitive proteins, such as Yes-associated protein (YAP). This suggests that microgravity disrupts mechanical signaling pathways critical for cellular regulation and bone homeostasis [37,38,39,40,41,42,43,44,45,46,47,48,49].

Mechanotransduction is a crucial process by which cells sense and respond to mechanical stimuli through interactions with the extracellular matrix. On Earth, proteins like YAP/TAZ translate mechanical forces into gene expression changes, modulating cell growth, differentiation, and survival. Under microgravity, the diminished activity of YAP/TAZ leads to varying outcomes in different cancers, such as reduced cell proliferation or increased invasiveness due to altered mechanosignaling. Understanding these differential responses could inform novel therapeutic strategies targeting these vulnerabilities [19,20,21,22].

Importantly, the application of compressive pressure partially restored YAP expression and increased pSMAD1/5/9 levels, demonstrating that biomechanical signals can reactivate essential molecular pathways, even under microgravity conditions. These findings underscore the potential of mechanical interventions to mitigate the adverse effects of microgravity on bone tissue [39,40,41,43].

The implications of these studies are significant. They not only advance our understanding of how microgravity influences cellular biology but also pave the way for developing therapeutic strategies to combat bone loss in both space environments and terrestrial patients with osteoporosis. Additionally, the creation of devices that simulate mechanical loads could benefit astronauts and individuals on Earth, emphasizing the critical role of biomechanics in maintaining bone health and cellular functionality in extreme conditions [39,40,41,43].

### 5.4. Kidney Cells

A recent study utilizing a microphysiological model of human renal proximal tubules investigated the effects of microgravity on the cellular ability to metabolize vitamin D and respond to human serum [44,45].

The findings revealed that short-term exposure to microgravity does not significantly alter essential biochemical pathways in renal cells, indicating that fundamental processes remain intact. In this experiment, renal proximal tubule epithelial cells were cultured in a microphysiological system and exposed to 2% human serum and vitamin D [25(OH)D3]. Biomarkers such as kidney injury molecule 1 (KIM-1) and interleukin 6 (IL-6) were measured to assess cellular responses [44,45,46].

Transcriptomic analyses, conducted via RNA sequencing, monitored vitamin D metabolites and key genes involved in its metabolism, including CYP27B1, CYP24A1, and CYP3A5 [44]. Surprisingly, no significant differences in vitamin D metabolism were observed between microgravity and terrestrial conditions. However, exposure to human serum elicited similar metabolic responses in both environments, characterized by metabolic reprogramming, alterations in bioenergetic processes, and the presence of a pro-inflammatory extracellular environment [44,45,46].

One of the most striking observations was the differential regulation of over 2000 genes after exposure to human serum, regardless of gravitational conditions. Affected pathways included the cell cycle, cytokine–cytokine receptor interactions, and chemokine signaling [45]. Notably, a marked reduction in the expression of genes associated with oxidative phosphorylation and fatty acid metabolism indicated diminished mitochondrial respiration, suggesting a metabolic shift in kidney cells [45].

These findings imply that kidney cells, when exposed to human serum, redirect their metabolism toward a proliferation-focused strategy, reducing reliance on traditional energy pathways. Concurrently, significant changes in the extracellular environment were observed, indicative of tissue remodeling. This phenomenon was supported by the overexpression of genes related to the extracellular matrix, including fibronectin (FN1) and transforming growth factor-beta-induced protein (TGFBI), as well as enzymes such as metalloproteinase 7 (MMP7), which are involved in remodeling and reorganizing the cellular environment. These adaptations may represent a cellular response to protein challenges in human serum or could signal underlying pathological processes [44].

In addition to bioenergetic and extracellular matrix alterations, broader metabolic reorganization signals were detected. Elevated cytosolic cholesterol levels, inferred from the repression of genes regulated by the SREBF2 factor, reflected shifts in intracellular metabolic balance. While these effects were consistent across microgravity and terrestrial conditions, their significance lies in demonstrating how human serum, as an external stimulus, can drive profound cellular changes independently of gravity.

These results reinforce the notion that certain fundamental processes in renal cells remain relatively stable under microgravity conditions during short-term exposures. However, they also highlight the need for further research into prolonged microgravity environments, where cellular adaptations may become more pronounced or clinically relevant [44,45].

The implications of these discoveries are significant for both space exploration and terrestrial medicine. In the context of spaceflight, the findings provide critical insights into how kidneys might adapt to microgravity, ensuring that essential functions such as filtration and blood compound regulation are maintained during extended missions. On Earth, these studies offer valuable perspectives on how kidney cells respond to metabolic and protein stress, potentially informing the development of advanced therapies for kidney diseases [44] (Figure 3).

### 5.5. Reproductive System Ovarian Follicles and Egg Quality

Simulated microgravity has provided valuable insights into how this unique environment impacts egg quality and ovarian follicle development. Ovarian follicles, which are critical for egg formation, rely on intricate interactions between granulosa cells and the egg to ensure proper maturation. However, the specific effects of microgravity on these interactions and the overall evolution of follicles have only recently begun to be understood [47].

In an experiment replicating microgravity conditions, ovarian follicles were cultured in a simulated system. The results indicated that while follicle growth and survival rates were not significantly affected and remained comparable to those observed under normal gravity conditions, the quality of the eggs released was markedly reduced [47,48,49].

Electron microscopy revealed alterations in the cellular structures responsible for communication between granulosa cells and the egg. These structural changes resulted in a loss of organization within the granulosa cells, disrupting the secretion of essential factors required for egg maturation. However, supplementing growth factors and antioxidants significantly improved egg quality, suggesting that the adverse effects of microgravity could be mitigated through targeted interventions [47,48,49].

Further analysis of the eggs highlighted deficiencies in the distribution and density of cortical granules, structures vital for maturation. Additionally, there was a notable reduction in the number of eggs capable of releasing the first polar body—a critical process in egg development. These deficiencies were linked to increased oxidative stress levels, which further contributed to the decline in egg quality [47,48,49].

Another key finding was the loss of polarity in granulosa cells, which inhibited the formation of cellular projections essential for interacting with the egg. This disruption impaired communication between granulosa cells and the egg, negatively affecting the maturation process. Moreover, microgravity altered the formation of microvilli in the egg, structures crucial for interaction with surrounding cells. Eggs cultured under microgravity conditions exhibited a significant reduction in both the number and size of microvilli, potentially limiting their ability to release factors essential for development [47,48,49] (Figure 3).

### 5.6. Cardiomyocyte Maturation and Functionality

Simulated microgravity has been shown to significantly enhance the maturation and functionality of cardiomyocytes derived from human pluripotent stem cells. One of the most notable improvements observed under these conditions is mitochondrial biogenesis, a critical process for cellular maturation [9,10,23,50]. Mitochondrial biogenesis enables cells to produce energy more efficiently, a key factor for the proper functioning of mature cardiomyocytes. Experimental data have demonstrated an increase in mitochondrial content, evidenced by the expression of specific mitochondrial proteins and an elevated ratio of mitochondrial DNA to nuclear DNA. These findings suggest that microgravity stimulates mitochondrial formation, improving the cells’ capacity to generate ATP, the primary energy source for cardiac function [6,50].

In addition to increased mitochondrial content, simulated microgravity enhances mitochondrial respiration. This improvement, evidenced by elevated maximal respiration levels and ATP production, ensures the energy supply necessary for cardiomyocyte contraction and relaxation. These observations highlight the potential of microgravity to promote metabolic maturation in cardiomyocytes, rendering them more functional and suitable for therapeutic applications [6,50].

Another critical aspect influenced by microgravity is calcium handling, a vital process for cardiomyocyte contraction and relaxation. Cells cultured under microgravity exhibited greater amplitude in calcium transients, reflecting enhanced calcium storage capacity in the sarcoplasmic reticulum, a structure crucial for regulating intracellular calcium concentrations. Efficient calcium handling is essential for effective cardiac function, as it enables stronger and more coordinated muscle fiber contractions. These findings indicate that cardiomyocytes cultured in microgravity are not only more metabolically advanced but also display improved functionality consistent with mature cardiomyocytes [6,50].

Simulated microgravity also positively affects the structural properties of cardiomyocytes. Observations include increased sarcomere length, the functional unit responsible for muscle contraction, and longer Z-disks, key structures for transmitting contraction forces. Additionally, nuclear eccentricity and diameter were increased, further indicating a higher degree of cellular maturation. These structural enhancements bring cardiomyocytes cultured in microgravity closer to the characteristics of fully functional human cardiac cells, reinforcing their ability to perform physiological functions effectively [6,50].

The duration of exposure to microgravity plays a pivotal role in maximizing these benefits. Recent studies suggest that a 7-day exposure period is optimal for promoting significant improvements in cardiomyocyte maturation. Prolonged exposures of 14 days, however, did not yield additional benefits, indicating a specific time window for maximizing the positive effects of microgravity. Optimizing culture conditions within this timeframe is essential to achieve the best outcomes [50,51].

Advances in utilizing simulated microgravity to enhance cardiomyocyte maturation and functionality open new avenues in regenerative medicine, particularly for treating cardiac diseases such as myocardial infarctions. The ability to produce high-quality, mature cardiomyocytes is critical for developing effective cellular grafts capable of integrating into damaged tissues and restoring cardiac functionality. These breakthroughs represent a significant step forward in biotechnology, with far-reaching implications for the treatment of cardiac pathologies [6,50,51] (Figure 3).

## 6. Impacts on Stem Cells and Cell Regeneration

Microgravity has provided significant insights into how this environment influences fundamental biological processes such as tissue regeneration and cell repair [52,53]. Research conducted in real spaceflights and terrestrial simulations has demonstrated that microgravity impacts protein expression and the regulation of cellular pathways critical for the growth and regeneration of various stem cell types. One of the most notable pathways affected is the Hippo pathway, which plays a central role in cell proliferation and tissue damage response. Under microgravity conditions, this pathway is temporarily deactivated, creating a favorable state for cell proliferation and tissue repair. These effects have shown particular promise in regenerating organs such as the heart, with potential applications extending to other tissues [53,54].

**Figure 4:**
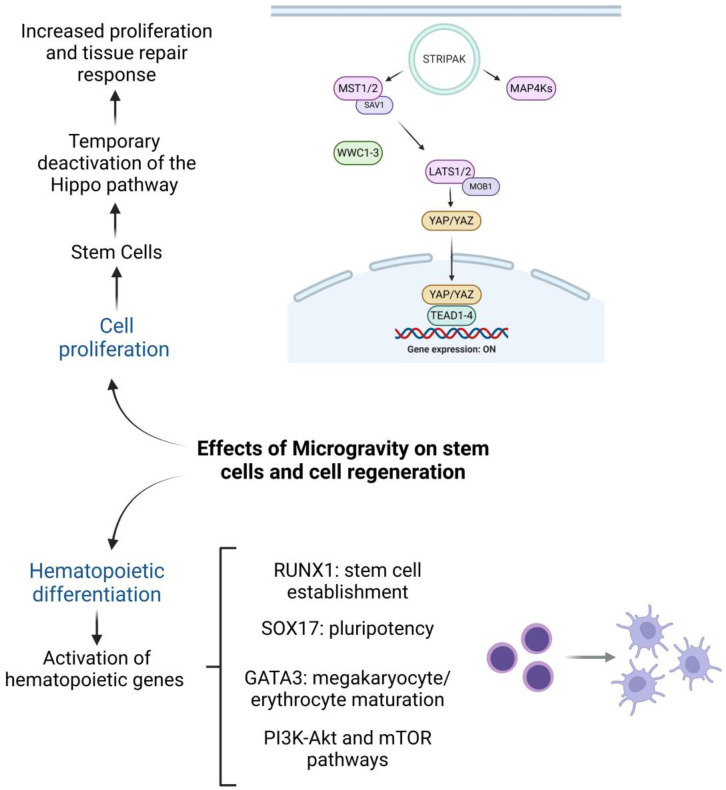
Microgravity effects on stem cell differentiation and regenerative potential. This figure illustrates key findings related to the effects of microgravity on stem cell behavior. Microgravity has been associated with enhanced cell proliferation, potentially contributing to tissue repair processes. Additionally, it leads to the temporary deactivation of the Hippo pathway, involving upstream regulators such as MST1/2, SAV1, WWC1-3, LATS1/2, and MOB1, which in turn promotes the nuclear translocation of YAP/TAZ. Furthermore, microgravity influences hematopoietic stem cell differentiation by activating specific genes and pathways, including *RUNX1*, which is involved in stem cell establishment, *SOX17*, which plays a role in maintaining pluripotency, and *GATA3*, which is crucial for megakaryocyte and erythrocyte maturation. The PI3K-Akt and mTOR pathways are also implicated in this process, with STRIPAK acting as a modulatory factor.

The replication of microgravity effects in terrestrial simulations opens new possibilities for exploring therapeutic treatments based on this phenomenon [17,52,53,54]. Findings suggest that microgravity could serve as a valuable tool for enhancing the regenerative capacity of stem cells without requiring invasive or complex technologies. Additionally, combining microgravity exposure with the use of certain drugs or microRNA manipulation—key regulators of gene expression—can amplify the activity of the Hippo pathway and YAP1 protein, further promoting tissue regeneration. These discoveries hold promise for treating heart diseases and repairing other damaged tissues and systems [53] (Figure 4).

Cells cultured under simulated microgravity using an RPM have shown significant alterations, enhancing our understanding of cellular responses to gravity loss. For example, thyroid carcinoma cells (FTC-133) formed three-dimensional spheroids, contrasting with their typical monolayer growth, and displayed changes in adhesion properties and internal organization consistent with spaceflight observations [23,24]. These morphological transformations involved cytoskeletal reorganization, particularly affecting actin filaments and microtubules, which are crucial for maintaining cellular integrity. Gene expression analysis revealed changes in genes related to cellular adhesion and proliferation, including vascular endothelial growth factor (VEGF), E-cadherin, and vimentin. Moreover, T lymphocytes exposed to RPM-simulated microgravity exhibited decreased activation, mirroring the effects observed in spaceflight experiments. These findings validate the utility of RPMs for ground-based research on physiological changes relevant to long-duration spaceflight [24].

### 6.1. Hematopoietic Differentiation

Simulated microgravity significantly enhances the differentiation of human pluripotent stem cells into hematopoietic lineages, offering a promising avenue for regenerative medicine and cell therapies [55,56,57]. Under microgravity conditions, hematopoietic stem cells demonstrate more efficient induction of endothelial hemogenic progenitors, leading to improved formation of functional three-dimensional structures. This results in a notable increase in active hematopoietic cells, optimizing both cell expansion and differentiation processes [55,56,57].

Transcriptomic analyses have identified the differential activation of thousands of genes involved in hematopoiesis, including RUNX1, SOX17, and GATA1. RUNX1 is essential for establishing hematopoietic stem cells, SOX17 for maintaining pluripotency and differentiation into hematopoietic lineages, and GATA1 for the maturation of erythrocytes and megakaryocytes. These gene expression patterns suggest that microgravity creates a favorable environment for optimizing hematopoiesis [55].

At the cellular level, microgravity influences DNA replication by promoting the expression of replication machinery genes such as helicases and polymerases. This enhances efficient cell division, a critical factor for hematopoietic stem cell proliferation and differentiation. Furthermore, microgravity modulates the cell cycle, facilitating transitions through key phases such as G1/S and G2/M, which are essential for cell proliferation [55,56,57].

A key discovery is the activation of the PI3K-Akt pathway, which regulates cell growth, survival, and metabolism. This pathway modulates proteins such as mTOR, which governs cell growth and metabolism, and Bad, which inhibits apoptosis, ensuring the survival of hematopoietic progenitor cells. By promoting cell proliferation and preventing programmed cell death, the PI3K-Akt pathway plays a pivotal role in the development of functional hematopoietic cells [56,57].

Microgravity also enhances three-dimensional cellular organization, improving interactions between cells and their microenvironment. These interactions are vital for forming hematopoietic niches that replicate physiological conditions more accurately. Cultures in microgravity favor the creation of three-dimensional spheroids, which support better differentiation and functionality of hematopoietic stem cells, optimizing the hematopoiesis process. This capability is particularly valuable for developing precise experimental models and innovative cell therapies [14,55,56].

Microgravity modulates hematopoiesis by influencing the differentiation, proliferation, and structural organization of hematopoietic stem cells (HSCs). Pluripotent stem cells exhibit enhanced differentiation potential, increased proliferation, and improved structural organization, as microgravity optimizes conditions for hematopoietic progenitor expansion [17,58,59,60,61,62]. This occurs through survival signaling, primarily via PI3K-Akt pathway activation, where increased Akt phosphorylation inhibits proapoptotic proteins such as BAD and enhances cell survival via antiapoptotic mechanisms linked to mTOR. Wnt/β-catenin signaling is also dynamically regulated in microgravity, promoting β-catenin accumulation in the cytoplasm and its nuclear translocation, activating genes essential for stem cell self-renewal and differentiation. Additionally, microgravity modulates Notch pathway interactions and transcription factors like RUNX1, crucial for hematopoietic lineage commitment. Structural rearrangements in hematopoietic niches under microgravity alter cell-to-cell interactions and adhesion molecule expression, facilitating the three-dimensional organization of hematopoietic clusters. On a physiological level, microgravity-induced metabolic adjustments enhance responsiveness to hypoxic stimuli, promoting hematopoietic maturation. These findings underscore the potential of microgravity-based systems to refine cell culture techniques and provide insights into spaceflight’s effects on human hematopoietic function [17,18].

### 6.2. Cell Proliferation and Regenerative Therapies

Microgravity significantly influences cell proliferation and functionality, representing a transformative advancement for biomedical research and regenerative medicine [63,64]. In studies with pancreatic islets and neural crest stem cells, exposure to microgravity notably enhanced cell proliferation, particularly in three-dimensional (3D) cultures. For instance, pancreatic beta cells exhibited a 45% increase in proliferation after two weeks of microgravity exposure compared to controls under normal gravity conditions [63,65].

Microgravity not only boosts cell growth but also modulates specific functions. For example, pancreatic beta cells cultured under microgravity showed a significant increase in insulin secretion, suggesting potential applications in developing therapies for metabolic diseases such as diabetes [66]. Furthermore, co-culturing stem cells with pancreatic islets under microgravity improved cell viability by 20%, highlighting its ability to create a favorable environment for cell development [63,67].

The formation of three-dimensional structures under microgravity is a critical aspect of these improvements. Unlike two-dimensional cultures, 3D models more accurately replicate human physiological conditions, allowing for complex cell–cell and cell–environment interactions essential for differentiation, proliferation, and functionality. Beta cells in 3D models not only proliferated more rapidly but also maintained enhanced functionality, as demonstrated by increased insulin secretion [63,66].

At the molecular level, microgravity appears to modulate key signaling pathways, such as PI3K-Akt and mTOR, which regulate cell survival, proliferation, and metabolism. While further studies are needed to confirm their specific roles in beta cells and neural crest stem cells, these findings underscore microgravity’s ability to influence fundamental processes and optimize cell culture conditions [63,65].

Microgravity offers a unique environment that enhances cell proliferation, viability, and functionality in 3D models, with direct applications in regenerative medicine. This environment not only promotes cell growth but also improves the functional quality of cells, which can significantly advance the development of cell therapies. For instance, pancreatic islets cultured under microgravity conditions could exhibit superior efficacy in transplants for diabetes patients, demonstrating increased proliferation and functional capacity, which could greatly enhance therapeutic outcomes [63,67].

## 7. Impact on Pathogen Virulence

Microgravity, whether experienced during spaceflight or simulated in terrestrial models, induces significant changes in the genetic expression and physiology of pathogens affecting both humans and plants. These changes have profound implications for astronaut health, space exploration, and the prevention of foodborne illnesses in space environments [15,68,69].

### 7.1. Increased Pathogen Virulence and Resistance

Under microgravity conditions, bacterial pathogens such as *Salmonella enterica* and *Escherichia coli* exhibit increased virulence, antibiotic resistance, stress tolerance, and biofilm formation. This is often accompanied by a reduced median lethal dose (LD50) in animal models. For instance, *Salmonella Typhimurium* cultured under simulated microgravity demonstrated fivefold higher lethality in mice compared to cultures grown under normal gravity. Additionally, these pathogens have been observed to evade and suppress plant innate immunity, enabling colonization of intracellular spaces. While these processes are also observed on Earth, it remains unclear whether microgravity enhances these capabilities or merely facilitates their study [15,16].

### 7.2. Molecular Mechanisms Driving Virulence

Microgravity alters extracellular nutrient transport and activates molecular pathways that enhance bacterial virulence. A key factor is low fluid shear, a physical stimulus that mimics microgravity environments and is also present in human microenvironments such as the gastrointestinal tract. Low fluid shear conditions favor the expression of stress tolerance and virulence-related genes, including those linked to the type III secretion system (TS3) and iron metabolism regulation. For example, *Salmonella Typhimurium* exposed to microgravity shows increased tolerance to acidic conditions and upregulation of genes controlled by Fur, a critical regulator of iron acquisition and virulence in enteric pathogens [15,68,69].

### 7.3. Effects on Biofilm Formation and Stress Response

Microgravity also influences the transport of oxygen and phosphates due to the absence of convection, replicating conditions similar to those in human infections. These environmental changes impact gene expression related to stress tolerance and virulence. For instance, *Escherichia coli* demonstrates enhanced biofilm formation and increased expression of genes responsible for host cell adherence and lesion formation, thereby amplifying its pathogenic potential. However, these effects are not universal; variations in results suggest that the impact of microgravity depends on the pathogen type, culture medium, and specific experimental conditions [15,16].

A recurring observation across multiple studies is the decreased expression of Hfq, a key regulatory protein involved in stress response and virulence. Hfq plays a critical role in the adaptation of both Gram-negative and Gram-positive pathogens under stress conditions, including microgravity. Understanding the regulatory mechanisms involving Hfq could provide insights into mitigating the risks of pathogen virulence during prolonged space missions [15,16].

Microgravity significantly affects interactions between human pathogens and plants, increasing bacterial virulence and compromising plant defense mechanisms. For example, pathogens under microgravity conditions demonstrate an enhanced ability to suppress plant immune responses, facilitating colonization and intracellular persistence [53,54,55,56,57]. These effects underscore the importance of investigating pathogen behavior in extreme conditions to develop strategies that mitigate health risks in space environments [15,16].

## 8. Discussion

Recent research demonstrates that microgravity significantly influences a wide array of biological processes, including immune responses, cell proliferation, and tissue regeneration. This unique environment disrupts key mechanisms such as mitochondrial biogenesis, liver metabolism, intestinal homeostasis, and stem cell differentiation, with profound implications for human health, particularly during prolonged space travel (Table 1).

Advances in understanding the effects of microgravity on stem cells, T cells, and immune function have created new opportunities for therapeutic innovations. Alterations in cellular biology under microgravity conditions have been shown to affect diseases such as cancer, osteoporosis, kidney dysfunction, and infertility. Additionally, changes observed in endothelial cell behavior and multi-organ homeostasis highlight microgravity’s potential to revolutionize regenerative medicine and deepen our understanding of human physiology in extreme environments.

Microgravity also holds significant promise for personalized medicine by facilitating progress in regenerative therapies, immune-based treatments, and approaches to managing chronic diseases. These findings emphasize microgravity’s transformative potential to reshape cellular biology and its application to contemporary medical challenges, both in space and on Earth.

As a defining characteristic of space environments, microgravity imposes profound and multidimensional effects on biological and physiological systems, challenging conventional notions of human adaptation. The near-total absence of gravitational forces disrupts numerous bodily processes, posing risks to health during and after space missions. Key effects include fluid redistribution, increased intracranial pressure, muscle atrophy, and calcium-related bone density loss. At the molecular level, microgravity alters gene expression, cell signaling, and mitochondrial function, with significant consequences for energy production and cellular health [37,38,39].

In the immune system, microgravity reduces T cell activity and suppresses immune responses, increasing susceptibility to infections. These effects highlight the necessity of mitigating immune dysfunction during space travel. Similarly, studies have documented changes in the human microbiome, with potential implications for astronaut health and nutritional strategies during prolonged missions.

Microgravity research has also led to technological and medical breakthroughs with applications beyond space exploration. For instance, the study of proteins and drug crystals under microgravity has produced purer and more stable structures, offering new therapeutic possibilities for chronic diseases. Additionally, materials research in space has contributed to the development of innovative compounds with applications in both space and terrestrial industries. These advancements enhance the safety and efficiency of space missions while delivering broad scientific and technological benefits [39,40].

Despite these advances, the complex effects of prolonged exposure to microgravity underline the need for further research, particularly in anticipation of missions to Mars and other distant destinations. Experimental models, such as space platforms and terrestrial simulators, remain indispensable tools for understanding the long-term impacts of microgravity and developing effective countermeasures [37,49].

The study of microgravity not only expands our understanding of how the human body adapts to extreme environments but also drives innovations with profound implications for science, medicine, and technology. By pushing the boundaries of what is known, this research establishes itself as a cornerstone for advancements that extend beyond space exploration, reshaping our perspectives on biology and human health [37,40].

## 9. Conclusions

Microgravity presents a unique and multifaceted challenge for human biology, profoundly influencing cellular systems and physiological processes. This environment, distinct from terrestrial conditions, disrupts key biological functions such as immune activity, cell differentiation, tissue regeneration, and mitochondrial biogenesis, thereby destabilizing homeostasis critical for human health.

Emerging research has highlighted the diverse effects of microgravity, including its impact on the immune system, where T cell function diminishes, increasing susceptibility to infections. Additionally, cancer cells exhibit altered behavior under microgravity, providing new insights into tumor progression and potential therapeutic approaches. These findings underscore the need for further investigation into how microgravity influences disease mechanisms and treatment responses.

Microgravity also affects the physiology of vital organs, such as the liver, kidneys, and bones, with implications for understanding tissue regeneration. Notably, stem cells exposed to microgravity demonstrate enhanced potential to produce mature, functional cells such as cardiomyocytes, offering transformative possibilities in regenerative medicine for repairing damaged cardiac tissue and other applications.

The continued exploration of microgravity’s effects is essential not only for safeguarding astronaut health during prolonged space missions but also for advancing therapeutic strategies on Earth. The ability of microgravity to induce unique biological changes offers unparalleled opportunities to innovate in regenerative medicine, cancer treatment, and the management of immune dysfunctions.

By leveraging microgravity as a research platform, medicine and biotechnology stand to gain transformative insights and novel applications, extending the boundaries of our understanding of human biology and driving the development of therapies that can improve health outcomes both in space and on Earth.

## Figures and Tables

**Table 1:** Summary of the effects of microgravity on different cells and tissues, including molecular pathways and pathogen behavior.

Cell/Tissue Type	Key Effects of Microgravity	Source	Reference
Macrophages	Imbalance between M1 and M2 polarization	Human	[1,2,58]
Dendritic Cells	↓Antigen presentation	Human Mouse	[1,58]
Neutrophils	↑Numbers but with ↓functionality	Human	[2,3,61]
B Lymphocytes	↓Proliferation and increased apoptosis	Human Mouse	[1,59,61]
NK Cells	↓Cytotoxic activity	Human	[1,59,70]
T Cells	↓Activation and proliferation	Human Mouse	[1,59,62]
Key Molecular Pathways	Dysfunction in RAS, ERK, and NF-κB pathways	Human	[1,4,59]
Cancer Stem Cells	↑Apoptosis	Cell culture of the following: Breast cancer Lung Cancer Gastrointestinal Cancer	[5,6,8]
Thyroid Cancer Cells	Low proliferation and growth	Human Mouse	[29,54]
Microvascular Endothelial Cells	Inhibition of proliferation	Human	[9,10,23]
Hepatic Cells	↑Lipid accumulation (lipotoxicity)	Rhesus Macaques Rodents	[34]
Intestinal Cells	Dysbiosis	Human	[34,36]
Bone Cells	Increased bone mass loss	Human	[41]
Kidney Cells	↓Oxidative phosphorylation and fatty acid metabolism genes (↓mitochondrial respiration, metabolic shift)	Human	[46]
Reproductive Cells	↓Egg quality	Mouse In Vitro Human Oocytes	[39,47,49]
Cardiomyocytes	↑Maturation and functionality	Human	[50,51,52]
Stem Cells	Temporary deactivation of the Hippo pathway (increased proliferation and tissue repair response)	Mouse Human	[53,54,55]
Hematopoietic Cells	↑Differentiation	Human Mouse	[55,56]
Cell Proliferation and Regenerative Therapy	↑Cell proliferation	Cell culture of the following: Pancreatic islets Neural crest stem cells	[63,66]
Pathogens	↑Virulence	*Salmonella enterica* *Salmonella Typhimurium* (in mice) *Escherichia coli*	[15,16,68,69]

↑: increase; ↓: decrease.

## Data Availability

Not applicable.
